# Histiocytoses and reactive proliferations of histiocytes: current state of the art and evolving concepts—a report from the joint CSHP-EA4HP-SH workshop 2024, Hefei, China

**DOI:** 10.1007/s00428-025-04096-4

**Published:** 2025-04-10

**Authors:** Falko Fend, Stefan Dirnhofer, Caoimhe Egan, Sophie Song, Zhe Wang, Xiaoqiu Li, Weiping Liu, Wenbin Xiao, Jean-Francois Emile, John Goodlad, Robert Lorsbach

**Affiliations:** 1 Institute of Pathology and Neuropathology and Comprehensive Cancer Center, Tübingen University Hospital, Liebermeisterstrasse 8, 72076 Tübingen, Germany; 2 Pathology, Institute of Medical Genetics and Pathology, University Hospital Basel, University of Basel, Basel, Switzerland; 3 Haematopathology & Oncology Diagnostic Service, Cambridge University Hospitals, NHS Foundation Trust, Cambridge, UK; 4 Department of Pathology and Laboratory Medicine, UCLA Health/David Geffen School of Medicine at UCLA, Los Angeles, CA USA; 5 Department of Pathology, Xijing Hospital and School of Basic Medicine, Fourth Military Medical University, Xi’an, People’s Republic of China; 6 Department of Pathology, Shanghai Cancer Center, Fudan University, Shanghai, People’s Republic of China; 7 Department of Pathology, West China Hospital, Sichuan University, Chengdu, People’s Republic of China; 8 Department of Pathology, Memorial Sloan Kettering Cancer Center, New York, NY USA; 9 Paris-Saclay University, Versailles SQY University, EA4340-BECCOH, Assistance Publique–Hôpitaux de Paris (AP-HP), Ambroise-Paré Hospital, Smart Imaging, Service de Pathologie, Boulogne, France; 10 Department of Pathology, NHS Greater Glasgow and Clyde, Glasgow, Great Britain; 11 Department of Pathology and Laboratory Medicine, Cincinnati Children’S Hospital Medical Center, University of Cincinnati, 3333 Burnett Avenue MLC 1035, Cincinnati, OH 45229 USA

**Keywords:** Hemophagocytic lymphohistiocytosis, Histiocytosis, Classification of histiocytic disorders, Joint workshop

## Abstract

Reactive and clonal proliferations of histiocytes (macrophages/dendritic cells) represent a broad spectrum of disorders, which can affect virtually any organ of the body. The clinical spectrum ranges from benign, localized and self-limiting manifestations to severe multi-system disease. Hemophagocytic lymphohistiocytosis (HLH) is a frequently life-threatening, systemic hyperinflammatory process triggered by massive cytokine release by activated, reactive macrophages. Familial and secondary forms of HLH are discerned. Histiocytoses are clonal inflammatory myeloid disorders characterized by proliferations of mature histiocytes/macrophages and dendritic cells with recurrent kinase-activating mutations which result in constitutive activation of the ERK signaling pathway. Although traditionally subclassified according to the phenotype of the lesional cells, the clonal cells can show significant plasticity, and the occurrence of mixed histiocytoses is increasingly recognized. This is in part due to their derivation from a myeloid progenitor cell and explains the frequent association with clonal hematopoiesis or overt myeloid neoplasms in adults. At the joint Workshop of the Chinese Society of Hematopathology, the European Association for Haematopathology and the Society for Hematopathology on histiocytic/dendritic cell proliferations, neoplasms, and their mimics in Hefei, China, April 2024, in sessions 1 and 2 a total of 8 cases of HLH, 9 cases of reactive histiocytic proliferations and 40 cases of histiocytoses were submitted and reviewed by the panel. The latter included cases of LCH, indeterminate cell histiocytosis, Erdheim Chester disease, juvenile xanthogranuloma, Rosai Dorfman disease, multicentric reticulohistiocytosis, ALK-positive histiocytosis, and mixed histiocytoses. The present report summarizes important findings and open questions arising from discussing the workshop cases.

**Supplementary Information:**

The online version contains supplementary material available at 10.1007/s00428-025-04096-4.

## Introduction

Clonal and reactive proliferations of histiocytes (macrophages and dendritic cells) represent a broad spectrum of mostly rare disorders, in which accumulations of cells of macrophage/histiocyte or dendritic cell lineage can occur in virtually any organ of the body and result in clinical symptoms through direct, lesional cell-related tissue damage and/or induction of inflammatory responses [1–3]. Recent advances in pathogenesis and molecular features significantly impacted our understanding and classification of these heterogeneous disorders.

Hemophagocytic lymphohistiocytosis (HLH) is a clinical syndrome characterized by uncontrolled, systemic inflammation that is life-threatening if clinically unrecognized or untreated. A cardinal feature of HLH is the systemic proliferation and accumulation of reactive histiocytes in various organs and tissues, including spleen, liver, and bone marrow, the latter two, together with cerebrospinal fluid, being the most commonly sampled tissues/fluids in patients with suspected HLH. In these anatomic sites, activated-appearing histiocytes are increased with a subset of hemophagocytic histiocytes that contain engulfed hematopoietic cells and platelets, contributing to the cytopenia characteristic of HLH. Aside from HLH, the accumulation/proliferation of reactive histiocytes may be encountered in a wide array of anatomic sites depending on the underlying clinical scenario. Such proliferations most often occur secondary to storage diseases, coexisting infectious or inflammatory disorders but may infrequently be associated with neoplasms. They may mimic a malignancy including true histiocytosis and/or obscure an underlying neoplasm.

Histiocytoses are rare disorders which can develop at any age and are characterized by the accumulation of mature cells of histiocytic (macrophage or dendritic cell) lineage in virtually any organ. Their clinical spectrum reaches from unifocal, self-limiting lesions with benign behaviour to life-threatening multisystem disease. Histiocytoses have long been regarded as chronic inflammatory disorders of unknown cause. Many were initially described as distinct clinicopathological syndromes, some of which were later grouped within a single entity with variable clinical manifestations, defined by the phenotypic characteristics of the lesional cells, such as in Langerhans cell histiocytosis (LCH) (Table 1) [4]. Recurrent pathogenic kinase mutations resulting in constitutive activation of the MAP kinase pathway have been detected in several different histiocytosis, confirming their clonal nature. These shared MAP kinase pathway abnormalities provide an opportunity for the use of novel therapeutic approaches in the treatment of these disorders that target activated kinases or inhibit downstream signalling pathways [5–10]. The presence of recurrent mutations, such as the *BRAF*^V600E^, has also facilitated clarification of the cell types involved in these disorders and correlation of the mutational status with prognostic features [11, 12]. Furthermore, the presence of *BRAF* mutations in both components of mixed histiocytoses, such as LCH and Erdheim-Chester disease (ECD) highlighted the plasticity of the clonal process [13]. In adults, the frequent association of histiocytoses with either overt myeloid disorders or clonal hematopoiesis suggests that many cases are manifestations of clonal stem cell disorders with significant skewing towards a histiocytic/dendritic cell differentiation [14–18]. Despite their clonality, the histiocytoses indeed exhibit features of inflammatory disorders, including spontaneous regression in a subset of cases and the recruitment of reactive inflammatory cells by the clonal population. These two phenomena are probably due to oncogene-induced senescence program in *BRAF-* or *RAS*-mutated cases and an accompanying senescence-associated secretory phenotype with increased secretion of IL- 1 and IL- 6 [3, 19].

**Table 1:** Clinicopathological features of common forms of histiocytosis

	Clinical features	Morphology	Phenotype	Genetics	Comments
Langerhans cell histiocytosis (LCH)	Predominantly childhood, M > F, broad clinical spectrum (unisystem, multisystem +/− risk organ involvement)	Variable number of LC with oval, grooved nuclei, eosinophils and multinuclear cells, old sclerotic lesions rare in LC	S100 +, CD1a +, Langerin (CD207) +, CD4 + Variable CD14, CD68 (paranuclear dot-like), low CD45	*BRAF* V600E 50% *MAP2K1* exon 2,3 10–20% rare *ARAF*, *CSF1R, NTRK* fusions and mutations	Frequent self-healing, especially solitary lesions; BRAF mutations and multi-organ involvement as risk factors
Indeterminate cell histiocytosis (IDCH)	Congenital self-healing form, classic presentation with multiple cutaneous lesions in adulthood	Mononuclear cells resembling LC, frequently ample cytoplasm	Defining: S100 +, CD1a +, Langerin (CD207) neg	*ETV3::NCOA2* specific (?) for adult IDCH, RAS/MAPK pathway mutations in IDCH associated with MN	1/3 of cases in adulthood associated with myeloid disorders
Erdheim Chester disease (ECD)	Non-LC histiocytosis of adults, characteristic long bone lesions, retroperitoneal (“hairy kindey”), and cardiovascular involvement	Lipid laden foamy macrophages, occasional Touton giant cells; difficult separation from reactive histiocyte accumulations	CD68 +, CD163 +, Lysozyme +, CD14 +, Fascin + FXIIIa +, S100 −, CD1a −, CD207 −	> 50% BRAF mutations (V600E) *MAP2K1*, *RAS*, *ARAF* mutations, rare *BRAF*, *NTRK* translocations	Frequent occurrence as “mixed histiocytosis” (60% with LCH), usually same mutation in all lesions with different phenotypes
Juvenile xanthogranuloma (JXG)	Classic form as single to multiple skin lesions with self-healing tendencies, < 5% systemic	Broad range of morphology with some lipidization and Touton type giant cells	CD68 +, CD163 +, CD4 +, CD14 +, FXIIIa +, S100 −/+, CD1a −, CD207 −	*BRAF* V600E and *MAP2K1* mutations in systemic forms	Systemic and CNS variants of JXG probably represent variants of ECD
Multicentric reticulohistiocytosis	Rare, characteristic clinicopathological syndrome with typical skin lesions and often severe arthritis	Mononuclear histiocytes with eosinophilic cytoplasm, no foam cells	CD68 +, CD163 +, CD4 +, CD11c +, FXIIIa +, CD1a −, CD207 −, S100 −	Data lacking, single case with *MAP2K1* mutation	Association with autoimmune disorders, malignancy, and hyperlipidemia
Rosai Dorfman disease (RDD)	classic sporadic RDD with massive bilateral cervical, self-limited LAP; > 40% with diverse extranodal manifestations	Large mononuclear histiocytes with large, round nuclei, emperipolesis; significant admixture of lymphocytes and plasma cells	S100 +, CD68 +, CD163 +, Cyclin D1 +, OCT2 +, Fascin +, CD1a-CD207 −	Sparse data; 30–50% of extracutaneous RDD with RAS/MAPK pathway mutations	May be difficult to discern from lymphoma and reactive disorders, familial forms, and association with autoimmune disorders
ALK + histiocytosis	Mostly young patients, broad clinical spectrum. 1a/b: Systemic form w/wo liver and BM; 2: single lesion; overall 50% CNS involvement	Broad spectrum of morphology (JXG-, RDD-like, spindled and epithelioid forms)	ALK +, CD68 +, CD163 +, S100 −/+, CD1a −, CD207 −, Oct2 +/−, CyclinD1 +	70% *KIF5B::ALK* fusions; *CTLC*, *TMP3*, *TFG*, *EML4* as other partners	Good response to ALK inhibitors; separation from ALK + inflammatory myofibroblastic tumor and other ALK + lesions may be difficult

The classification of the histiocytoses has evolved significantly since the discovery of driver alterations and an increased understanding of the plasticity of macrophages and dendritic cells. Currently, three different classification systems are in use for histiocytic disorders including high grade malignancies derived from macrophages and dendritic cells. Both the 5th Edition of the WHO classification and the International Consensus Classification (ICC) are primarily based on the cell of origin and its phenotypic characteristics or defining molecular alterations, as in the new category of ALK-positive (ALK +) histiocytosis, and show only minor differences in systematic grouping [20–22]. The revised classification of the Histiocyte Society published in 2016 takes a distinct approach with clinicopathological groupings, recognizes the interrelationship of LCH and Erdheim-Chester disease, and also includes hemophagocytic lymphohistiocytosis (HLH), a reactive histiocytic disorder [23].

The joint Workshop of the Chinese Society of Hematopathology (CSHP), the European Association for Haematopathology (EA4HP) and the Society for Hematopathology (SH) on histiocytic (macrophage/dendritic cell) proliferations, neoplasms, and their mimics was held in Hefei, China, April 13–14, 2024. The aim of the workshop was to review the clinico-pathological spectrum of these disorders, discuss diagnostic approaches, issues of classification, and the handling of unusual and borderline cases, which represented a significant fraction of the submitted cases, given the nature of a workshop. In this manuscript, cases presented in sessions 1 and 2, dealing with reactive histiocytic proliferations, HLH, and histiocytoses, respectively, and the conclusions and remaining areas of discussion are summarized.

## Hemophagocytic lymphohistiocytosis and reactive proliferations of histiocytes

There are two major subtypes of HLH. Familial HLH (F-HLH), also referred to as genetic or primary HLH, is caused by predisposing mutations in one of several genes that impair cytotoxic granule-mediated elimination of target cells by activated cytotoxic T-cells and NK-cells due to either defective biogenesis or impaired delivery of cytotoxic granules by these effector immune cells [24–26]. Biallelic mutations in 3 genes, *PRF1*, *STXBP2*, or *UNC13D*, account for approximately 70% of P-HLH cases [27]. F-HLH typically manifests in infancy or early childhood as episodic, life-threatening systemic inflammation. While treatment with corticosteroids and etoposide is used for acute HLH flares, bone marrow transplantation is the optimal curative therapy for most genetic subtypes of HLH [28]. Secondary HLH (S-HLH) is pathogenetically less well understood, is likely overdiagnosed and needs to be separated from processes simulating HLH. Therefore, use of the term “secondary HLH” is discouraged by some authors [25]. However, like F-HLH this syndrome is caused by dysregulated T-cell activation with resulting systemic macrophage activation [29]. As implied by its designation, systemic hyperinflammation in S-HLH is triggered by a broad array of underlying disorders, including malignancy and infectious and autoimmune etiologies [29]. While S-HLH due to infectious causes may occur at any age, S-HLH associated with malignancy or autoimmune disorders occurs primarily in adults. Distinction between primary and secondary HLH is critical, especially in older children in whom both disorders may be encountered, because successful treatment of S-HLH is predicated on identification and appropriate treatment of the underlying disorder [30]. Common clinical features of HLH include fever, cytopenias, and hepatosplenomegaly. Laboratory studies typically show hyperferritinemia and hypertriglyceridemia, hypofibrinogenemia, increased levels of soluble IL2 receptor (sIL2R) and CXCL9, and low or absent NK-cell activity [24]. Several of these clinical and laboratory parameters are included in the HLH- 2004 diagnostic algorithm [31]; however, these criteria do not reliably distinguish primary from secondary HLH, and thus, it is imperative to evaluate for underlying malignancy or infectious etiologies in patients lacking HLH-associated mutations [30].

**Fig. 1:**
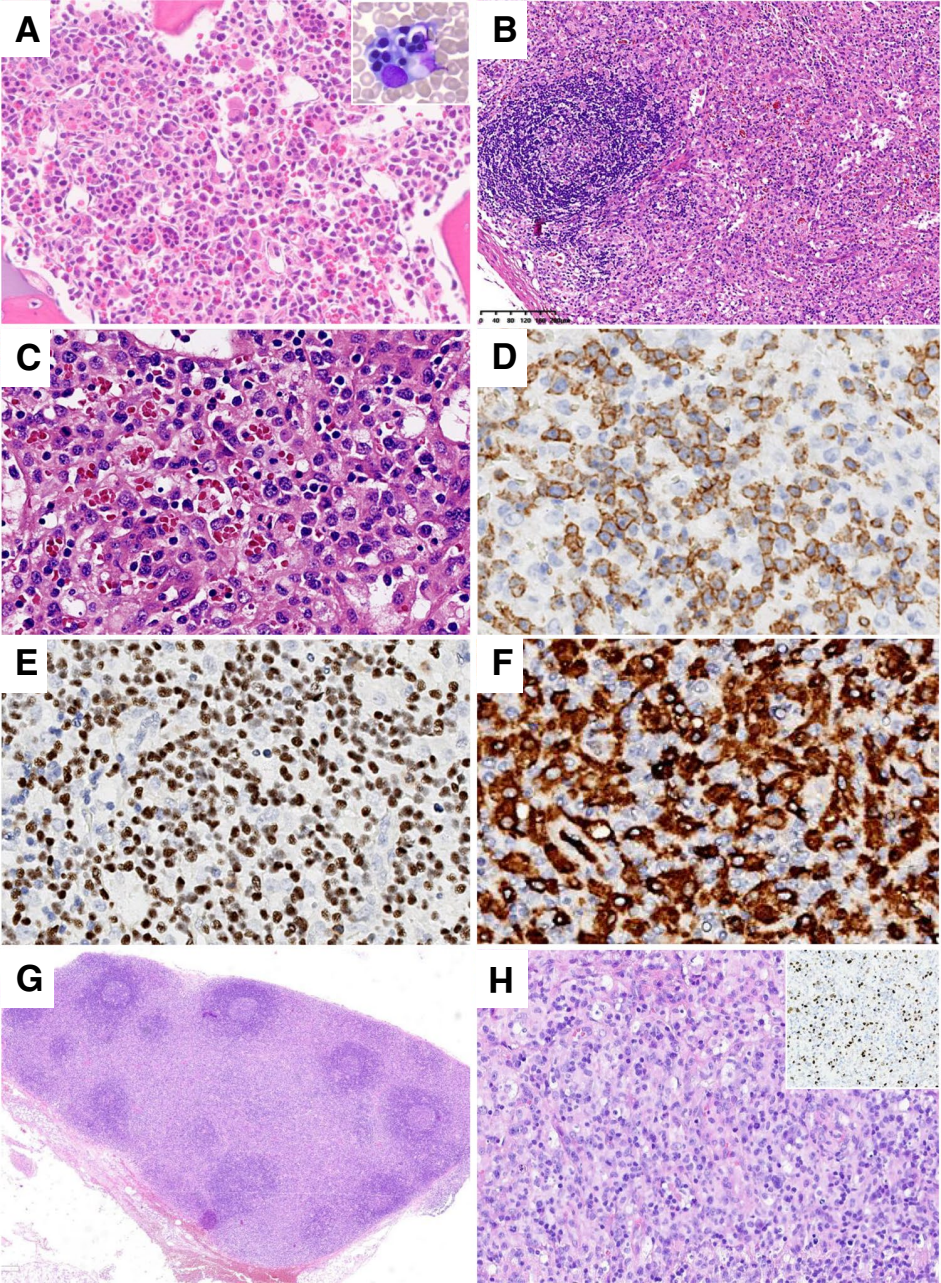
Hemophagocytic lymphohistiocytosis. **A** Bone marrow findings in familial HLH with homozygous mutations in *STXBP2* (case 221,618, S. Sirotnikov). Histiocytic hyperplasia is evident in the H&E section, with many hemophagocytic forms. Hemophagocytic histiocytes can be inconspicuous in H&E sections, particularly when present at low frequency. In such cases, CD163 immunohistochemistry facilitates the identification of hemophagocytic forms. The accompanying aspirate smear contains frequent hemophagocytic histiocytes containing nucleated erythroid progenitors (**A**, inset). **B**–**F** SETLC (case 221,571, J. Huang). **B** Lymph node showing marked paracortical expansion at low magnification. **C** Medium- and large-sized lymphoid cells are present with mild cytologic atypia are present within the paracortex, most of which are CD8 T-cells (**D**) and positive for EBV-EBER by in situ hybridization (**E**); these cells are also positive for CD2 and CD3 with partial loss of CD5 and CD7 (not shown) and coexpression of TIA1 and granzyme B (not shown). **F** Abundant hemophagocytic histiocytes admixed with the lymphoma cells are highlighted by CD163 immunohistochemistry. **G**, **H** HLH associated with primary EBV infection (case 215,254, Z. Chen). **G** There is paracortical expansion comprised of a polymorphous population of T-cells and somewhat fewer B-cells with less atypia than in case 221,571 (**H**). Frequent EBV-EBER positive cells are present (**H**, inset)

Two cases of F-HLH were submitted to the workshop. Case 221,618 (S. Sirotnikov) was a 2-month-old, full-term male infant who presented with fever and splenomegaly with pancytopenia, hyperferritinemia, hypertriglyceridemia, and elevated sIL2R levels; of note, EBV and CMV studies were negative. Bone marrow examination revealed prominent histiocytic hyperplasia with hemophagocytosis (Fig. 1A). A presumptive diagnosis of F-HLH was confirmed by next generation sequencing studies which identified homozygous mutations in *STXBP2* (c.1463 C > T,p.Pro488Leu). Case 221712 (M.Klimkowska), which similarly harbored *STXBP2* mutations, was notable for the patient developing HLH as a teenager and for subsequent development of classic Hodgkin lymphoma [32]. Atypical presentations of F-HLH, including older age at onset, have been associated with hypomorphic mutations in *STXBP2* and other F-HLH associated genes, highlighting the importance of thorough genetic evaluation in all patients with HLH, including adults [33, 34].

Six cases of non-hereditary-HLH were submitted to the workshop, three of which were associated with coexisting malignancy (215,644, Z. Hosseinzadeh; 221,571, J. Huang; and 224,361, N. Tangnuntachai) and the remaining 3 with underlying infection (case 215,254, Z. Chen, EBV; case 224,362, N. Tangnuntachai, dengue; and case 224,430, P. Bulterys, tuberculosis). All malignancy associated cases had underlying lymphoma, including diffuse large B-cell lymphoma (case 215,644), systemic EBV-positive T-cell lymphoma of childhood (SETLC; case 221,571), and low-grade B-cell lymphoma (case 224,361). Hematologic neoplasms are the most common type of HLH-associated malignancy. Although HLH has been reported in association with virtually all hematologic malignancies, it is most strongly associated with T-cell lymphomas, including ALK-positive anaplastic large cell lymphoma, subcutaneous panniculitis-like T-cell lymphoma, and other subtypes of peripheral T-cell lymphoma.

Two cases (cases 215,254, EBV positive HLH, Z. Chen; case 221,571, SETLC, J. Huang), both in young children, illustrate the diagnostic challenges in distinguishing between HLH secondary to EBV infection and that occurring secondary to SETLC. Lymph node biopsies from both cases were similar in appearance and showed marked paracortical expansion by atypical medium to large-sized CD8 positive lymphoid cells that co-expressed cytotoxic T-cell antigens, were EBV-EBER positive, and showed high proliferative activity as assessed by Ki- 67 staining (Fig. 1B–H). Both cases showed prominent histiocytic hyperplasia with hemophagocytosis. In both cases, the initial diagnostic impression was that of SETLC. Molecular studies in both revealed clonal T-cell receptor rearrangements, and no pathogenic mutations were identified by HLH NGS studies in either case. Both patients satisfied criteria for HLH and were treated per the HLH- 94 regimen. One patient (case 215,254) had a full recovery with no evidence of recurrent HLH or any lymphoproliferative disorder with 44 months of follow-up, whereas the second patient (case 221,571) had fulminant disease that was unresponsive to therapy and died 45 days after disease onset. HLH is a common feature of systemic acute EBV infection, systemic chronic active EBV disease and SETLC, and distinction between these EBV-associated lymphoproliferative disorders (LPDs) is often challenging using currently available diagnostic tools [35]. In all 3 disorders, lymphoid cells with mild cytologic atypia may be present in bone marrow and lymph node, and clonal T-cell populations may be detected [36–38]. An abnormal karyotype and pathogenic mutations appear to reliably distinguish SETLC from the other LPDs; however, cytogenetic aberrations are detected in only a minority of SETLC cases, and data on the mutational profile are sparse [38, 39].

Reactive histiocyte and dendritic cell hyperplasia may be present within lymph nodes in a variety of clinical settings. In case 214,477 (M. Chen), axillary lymphadenopathy developed following vaccination for COVID 19. Biopsy of this lymph node showed marked paracortical expansion with a prominent proliferation of paracortical Langerhans cells. Distinction from nodal involvement by LCH was facilitated by the typical paracortical localization of the Langerhans cells (LC) (versus the sinusoidal distribution characteristic of LCH) and the negativity of these cells for both cyclin D1 and mutant-specific BRAF by immunohistochemistry. A variety of histiocytic proliferations have been reported following vaccination for COVID 19, including Langerhans cell hyperplasia, proliferations with features of Kikuchi-Fujimoto disease and necrotizing granulomatous inflammation, as well as florid follicular hyperplasia [40–42]. Clinically evident lymphadenopathy develops on average about 2 weeks following immunization [40]. At cutaneous sites, histiocytic/dendritic cell proliferations may develop secondary to chronic dermatoses, mimicking LCH. In such cases, thorough immunophenotypic and molecular characterization aids in distinction of reactive dendritic cell proliferations from *bona fide* LCH, in which the lesional cells express cyclin D1 and frequently harbor the *BRAF* V600E mutation.

**Fig. 2:**
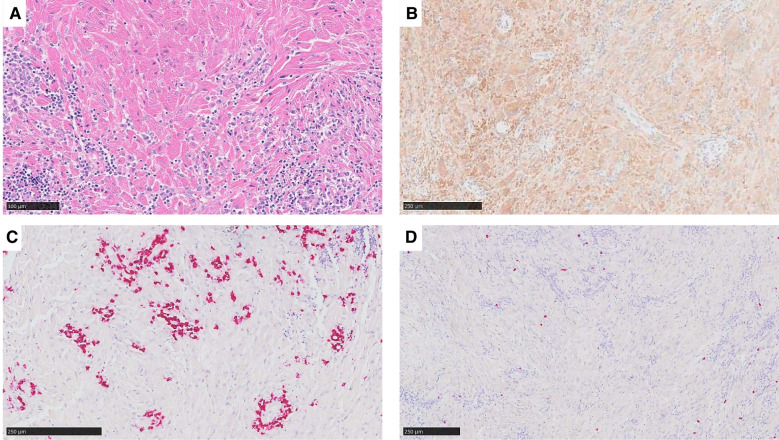
Crystal-storing histiocytosis. Biopsy of a “tumor-like” stenosis in the left main bronchus (case 214,325, X. Ge). **A** In the H&E section, there is a proliferation of spindle to epithelioid histiocytes with abundant eosinophilic, fibrillary cytoplasm, imparting a striated muscle-like appearance. **B** CD68 immunohistochemistry confirms the histiocytic phenotype of these cells. **C** Adjacent to these histiocytes with crystalline-like cytoplasmic inclusions, there is a B-cell lymphoplasmacytoid proliferation with clonal restriction for kappa light chain leading to the diagnosis of an extranodal marginal zone lymphoma (MALT – lymphoma); **D** lambda light chain stains few reactive plasma cells

Several cases of reactive proliferation of histiocytes associated with a neoplasm were submitted to the workshop. Three cases were classical examples of crystal-storing histiocytosis (CSH). This rare disorder is characterized by intra-histiocytic accumulation of crystallized immunoglobulins, usually monoclonal light chain kappa. It can be localized or systemic and is a manifestation of an underlying lymphoproliferative disorders (LPD) in most cases (80–90%), predominantly B-cell neoplasms such as multiple myeloma, lymphoplasmacytic lymphoma, or MALT-lymphoma [43]. Case 214,325 (X. Ge) was an example of localized (left bronchus) CSH in a patient with pulmonary MALT-lymphoma (Fig. 2). In case 214,900 (E. Johnson), the CSH mimicked pseudo-Gaucher cells and obscured the underlying multiple myeloma. No associated LPD was found in case 212,247 (Y. Li), which was diagnosed as idiopathic pulmonary CSH.

Dr. Saglietti (case 220,548) submitted a tenosynovial giant cell tumor (TSGCT) with a fusion transcript *CSF1::CD101*. This results in upregulation of CSF1 expression in the lesional cells. Via a so-called «landscape effect» CSF1 mediates proliferation of CSF1R-expressing macrophages and their precursors, which can “overgrow” the tumor cells [44].

A very complex case with a long clinical history was submitted by Dr. Croci (case 221,331). It was a myeloid/lymphoid neoplasms with eosinophilia (MLNE) and tyrosine kinase gene fusion (*ETV6::SYK*) detected in peripheral blood. The patient developed numerous cutaneous plaques consisting of a bland histiocytic proliferation, probably clonally related to the MLNE.

## Histiocytoses

### Langerhans cell histiocytosis

LCH is the prototypical disorder of this group and is defined by the proliferation of clonal Langerhans cells. Although it is primarily a childhood disorder with a mild predominance in males at an annual incidence of 5–9 cases/10^6^, LCH can occur throughout adulthood [18]. The clinical spectrum ranges from indolent solitary lesions to life-threatening systemic disease. The most commonly involved organs are bone (80%), skin (33%), hypophysis (15–25%), and lymph nodes, as well as spleen, liver (approx. 15% each), the latter 3 representing risk organs [2, 3, 45]. The frequency of lesional cells with the characteristic immunophenotype (CD1a +, CD207/Langerin +) varies considerably, as does the extent and composition of the background population with eosinophils, histiocytes and lymphoid cells. Since the seminal detection of the *BRAF*^V600E^ mutation in 50% of LCH cases overall [5], the spectrum of molecular alterations has been further elucidated. *BRAF*^V600E^ negative cases show mutually exclusive mutations in *MAP2 K1* (10–20%), other RAS-MAPK pathway genes, variant *BRAF* mutations and fusions involving *BRAF*, *NTRK*, or *CSF1R.* Somatic mutations are not detected in a minority of cases (10–20%) [6, 7, 10, 45–47]. *BRAF*^V600E^ cases show more frequent risk organ involvement, recurrences and development of neurodegenerative disease, the latter a rare complication of LCH [12]. Using mutations as clonal marker, studies examining different hematopoietic cell types in few cases suggested that the presence of *BRAF*^V600E^ in self-renewing hematopoietic progenitors results in disseminated high-risk disease, whereas restriction to more differentiated committed myeloid precursors or tissue-resident myeloid populations is associated with multifocal or unifocal low-risk LCH [11].

**Fig. 3:**
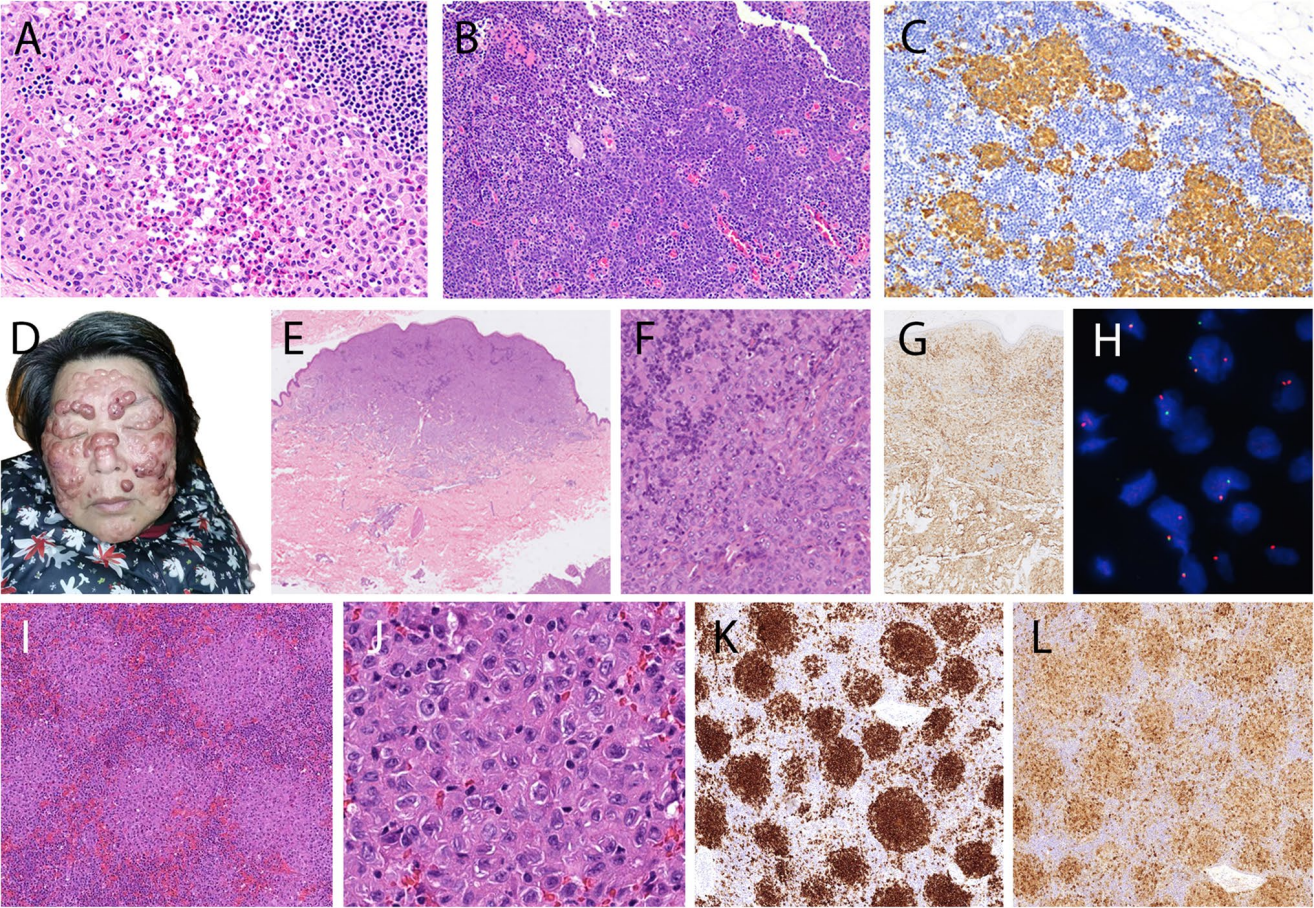
Langerhans cell histiocytosis (LCH) and indeterminate cell histiocytosis (IDCH). **A**–**C** Case 215,643 (X. Ni) LCH and metastatic nasopharyngeal carcinoma (NPC) in cervical lymph node. **A** LCH focus with typical LC aggregate and eosinophils. **B** Metastatic NPC focus, which showed positivity for cytokeratin, p40 and EBERs +. **C** Strong CD207 staining of LCH cells. **D**–**H** Case 221,640 (H. Li) IDCH. **D** Patient with leonine facies. **E** Low power view shows dense dermal infiltrate. **F** High power view shows a monotonous proliferation of cells with moderate eosinophilic cytoplasm and oval nuclei. **G** Homogenous CD1a expression, CD207 remained negative. **H** Demonstration of *NCOA2* break by FISH. **I**–**L** Case 220,829 (D. Laszko) IDCH in lymph nodes in patient with myelodysplastic syndrome. **I** Low power view shows strikingly nodular infiltrate of IDCH. **J** High power view shows moderate eosinophilic cytoplasm and irregular, cleaved nuclei. **K** Expression of CD1a and **L** S100

Seven cases of LCH were submitted to the workshop, all of them occurring in adult patients (range: 28–68 years). Two cases represented classic LCH of the bone and the lymph node, respectively, highlighting the fact that these typical manifestations are not restricted to children and young adults. Case 221,278 (S. de Pew), a 68-year-old male with multifocal lytic and lytic-sclerotic bone lesions and hypophyseal involvement was considered to represent a case of mixed histiocytosis (LCH and ECD) due to the presence of foamy histiocytes lacking CD1a and CD207 in several bone biopsies, with foci of true LC differentiation in only one of them. Three cases of LCH presented in the immediate vicinity of solid tumor manifestations, and an unusual case exhibited involvement of an arterial aneurysm in addition to lymphadenopathy, raising a differential diagnosis of exuberant reactive LC proliferations (Fig. 3A–C). However, the detection of the *BRAF*^V600E^ mutation in two of these cases, in accordance with published data, confirmed a clonal process and could indicate that clonal LC cells migrate to areas of inflammatory or immune reactions [48]. Of note, LCH in solid tumors likely represents a phenomenon distinct from those histiocytoses which develop in association with hematological neoplasms, mostly mature B cell lymphomas, in which the histiocytosis and lymphoma are usually clonally related, reflecting so-called transdifferentiation [49].

### Indeterminate dendritic cell histiocytosis (IDCH)

IDCH is distinguished from LCH by the lack of CD207 expression, but positivity for CD1a and S100 [50–52]. IDCH encompasses distinct clinical manifestations. Case 214,287 (A. Bonometti), a girl of four months at the time of diagnosis, but with lesions already present at birth, represented a good example of congenital self-healing IDCH with multiple skin lesions with spontaneous regression, similar to congenital cutaneous LCH. Case 221,640 (H.Li) was a 54-year-old female with multiple skin tumors, in which a *ETV3::NCOA2* fusion was demonstrated. This fusion has been described previously in several cases of adult cutaneous IDCH without accompanying myeloid neoplasm, thus possibly presenting a pathognomonic molecular feature for this rare entity [53], but is present in less than 20% of IDCH (Fig. 3D–H). The most frequently mutated gene in IDCH is *KRAS* (41%), contrasting with LCH [52]. Two further cases were associated with underlying myeloid neoplasms (primary myelofibrosis and MDS, respectively) and exhibited typical myeloid-type mutations in addition to a *KRAS* and *BRAF* mutation, respectively, resembling published similar cases (Fig. 3I–L) [54]. Underlying myeloid neoplasms are present in approximately 40% of adult IDCH cases, indicating that they probably represent specific outgrowths of a hematopoietic stem disorder [52].

### Erdheim-Chester disease and mixed histiocytosis

ECD is a non-Langerhans cell histiocytosis mainly occurring in adult patients with male predominance and a median age at diagnosis of 50–60 years. Symmetric, diaphyseal, and metaphyseal bilateral cortical osteosclerosis of long bones is present in more than 95% of patients, and cardiovascular involvement in > 50% [55–57]. Retroperitoneal involvement with “hairy kidney sign” on imaging due to infiltration of perirenal fat, CNS, and hypophyseal involvement and periorbital xanthelasmata are additional characteristic manifestations. Histologically, ECD shows accumulations of CD163 + and CD1a and S100 negative histiocytes with frequently foamy cytoplasm. In the absence of, or without knowledge of characteristic radiological findings, diagnosis requires a high level of suspicion, and many patients undergo repeat biopsies over an extended period of time before a diagnosis is achieved. The detection of a *BRAF*^V600E^ (present in more than 50% of cases) or other MAPK pathway mutations can help to corroborate the diagnosis. Of note, the variant allele frequency is often quite low (< 10%) and requires sensitive detection methods [58]. Fifteen to twenty percent of ECD patients show mixed histiocytosis, most commonly (60%) in association with LCH. Clonal hematopoiesis is frequent, and 10% have a co-occurring myeloid neoplasm [2, 15–17, 46, 57].

**Fig. 4:**
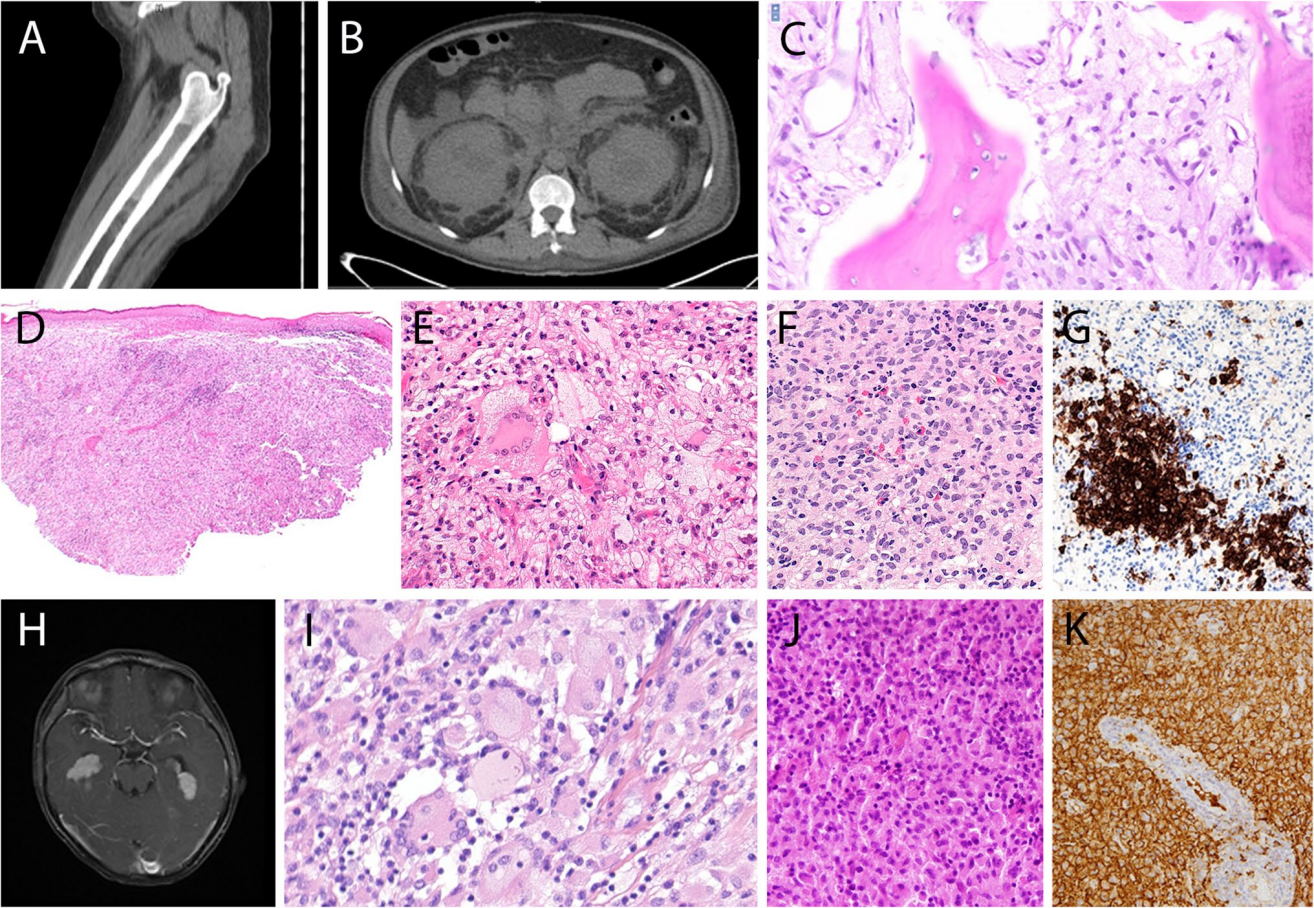
Erdheim Chester disease (ECD), mixed histiocytosis, and systemic “juvenile xanthogranulomatosis” (JXG). **A**–**B** Case 221,386 (D. Soliman) ECD. **A** Typical sclerotic foci in long bone. **B** CT scan of abdomen with typical “hairy kidney” sign caused by infiltration of perinephric fat. **C** Case 213,913 (C.L. Cheng), mixed histiocytosis with ECD and LCH. ECD lesion in tibial biopsy with marrow infiltration by foamy histiocytes. **D**–**G** Case 221,365 (J. Goodlad), mixed histiocytosis with ECD and LCH. **D**, **E** Submammary skin biopsy with foamy histiocytes and occasional Touton giant cells. **F** LN infiltrate by Langerhans cells with **G** CD1a expression. BRAF V600E was detected in both lesions. **H**–**K** So-called systemic JXG with CNS involvement better classified as non-LC histiocytosis. **H**, **I** Case 221,572 (S. Yang). **H** MRI scan (T1 enhanced) of the brain showing bilateral intraventricular masses. **I** Proliferation of histiocytes with pale cytoplasm and multinucleated cells, occasional emperipolesis. This case in an 8-year-old male showed a BRAF V600E mutation. **J**, **K** Case 221,412 (Z. Fan), 37-year-old male. **J** Monotonous proliferation of mononuclear histiocytes with K. strong CD163 expression

Five cases of ECD were submitted to the workshop, of which three showed features of a mixed histiocytosis, with LCH in two of them (Fig. 4C–G). Characteristic diffuse long bone lesions were present in all cases. 221,386 (D. Soliman, Fig. 4A–B) was a prototypical ECD case of a 60-year-old male with progressive erythematous skin patches, retroperitoneal fibrosing disease with bilateral hydronephrosis, severe anemia and hypermetabolic activity in long bones and bone marrow, and perinephric fat by PET CT. After several previous biopsies were interpreted as non-specific inflammation, bone marrow biopsy demonstrated extensive accumulations of frequently multinucleated histiocytes with signs of hemophagocytosis and presence of *BRAF*^V600E^ mutation, establishing the diagnosis. Case 221,365 (J. Goodlad) was a 46-year-old female with orange-colored submammary skin plaques of long duration. PET CT showed multifocal lymphadenopathy, widespread soft tissue involvement including retroperitoneum, and increased uptake in sacrum and long bones. Both lymph node and skin biopsies showed extensive accumulations of foamy histiocytes with interspersed, discrete nodules of CD1a +/CD207 + Langerhans cells, establishing the diagnosis of mixed histiocytosis (Fig. 4D–G).

An unusual case (214,289, A. Bonometti) revealed various phenotypes in different lesions, including ECD, LCH and Rosai-Dorfman-Destombes disease (RDD), all sharing an identical *MAP2 K1* mutation and finally progressing to AML. In summary, the workshop cases highlighted some of the classic features of ECD, as well as the common occurrence of mixed histiocytoses in this group.

### Juvenile Xanthogranuloma (JXG) and multicentric reticulohistiocytosis

Classic JXG is a non-Langerhans cell histiocytosis of infancy and childhood primarily manifesting in the skin followed by soft tissues and exhibits benign behavior with frequent self-healing [59]. Histologically, JXG shows proliferations of histiocytes with variable morphology and frequent interspersed Touton giant cells. They express CD4, CD68, CD163, factor XIIIa, and occasionally S100, but lack CD1a and CD207. Cutaneous JXG shows only few of the recurrent genetic alterations encountered in other disorders. Of note, cases of solitary ALK + histiocytosis of the skin are morphologically and immunophenotypically indistinguishable from JXG.

In contrast, cases of intracranial histiocytosis with JXG morphology have been shown to frequently contain *BRAF* mutations, and it has been proposed that these, as well as systemic cases, might represent a different entity, possibly childhood cases of ECD [60, 61]. Of note, cases from the JXG family with ERK pathway mutations are classified within the “L” group in the Histiocyte Society classification [23].

Four cases were submitted to the workshop under the heading JXG or xanthoma disseminatum. Cases 221,412 (Z.Fan) and 221,572 (S. Yang) were male patients of 8 and 37 years of age, respectively, with multifocal brain lesions and lack of other sites of involvement (Fig. 4H–K). Both showed accumulation of histiocytes and interspersed giant cells and expression of histiocyte markers, with additional S100 positivity in the adult patient. A *BRAF*^V600E^ mutation was identified in the childhood case, whereas the adult case lacked mutations in MAPK pathway genes. The panel considered both cases examples of non-Langerhans cell histiocytosis, with relationship to ECD and concluded that isolated CNS disease with JXG-like morphology and phenotype should not be lumped together with conventional cutaneous JXG.

Case 213,991 (J. Cheng) was a 12-year-old male with disseminated cutaneous JXG-like lesions appearing during consolidation treatment for B-lymphoblastic leukemia. The bone marrow biopsy also showed accumulations of histiocytes, and molecular examination demonstrated a *KRAS* G12D mutation; the ALL was not available for sequencing. This case is reminiscent of similar cases in the literature, in which either a transient monocytic differentiation during therapy or JXG-like cutaneous efflorescences have been described following acute lymphoblastic leukemia; for some of these cases, a common clonal origin has been described. These cases may exhibit atypical histology and bad prognosis [62].

**Multicentric reticulohistiocytosis** is a characteristic clinicopathological syndrome with multifocal papular skin lesions composed of histiocytes without xanthomatous changes, occasional involvement of mucous membranes and destructive arthritis. It occurs predominantly in females and is commonly associated with autoimmune disorders or malignancy [63–65]. Coalescent papules and nodules forming plaques with cobblestone appearance on the back of the hands and periungual papules giving a “coral bead” appearance are considered pathognomonic. Since the histological picture is nonspecific, the characteristic clinical features are necessary to reach the correct diagnosis.

**Fig. 5:**
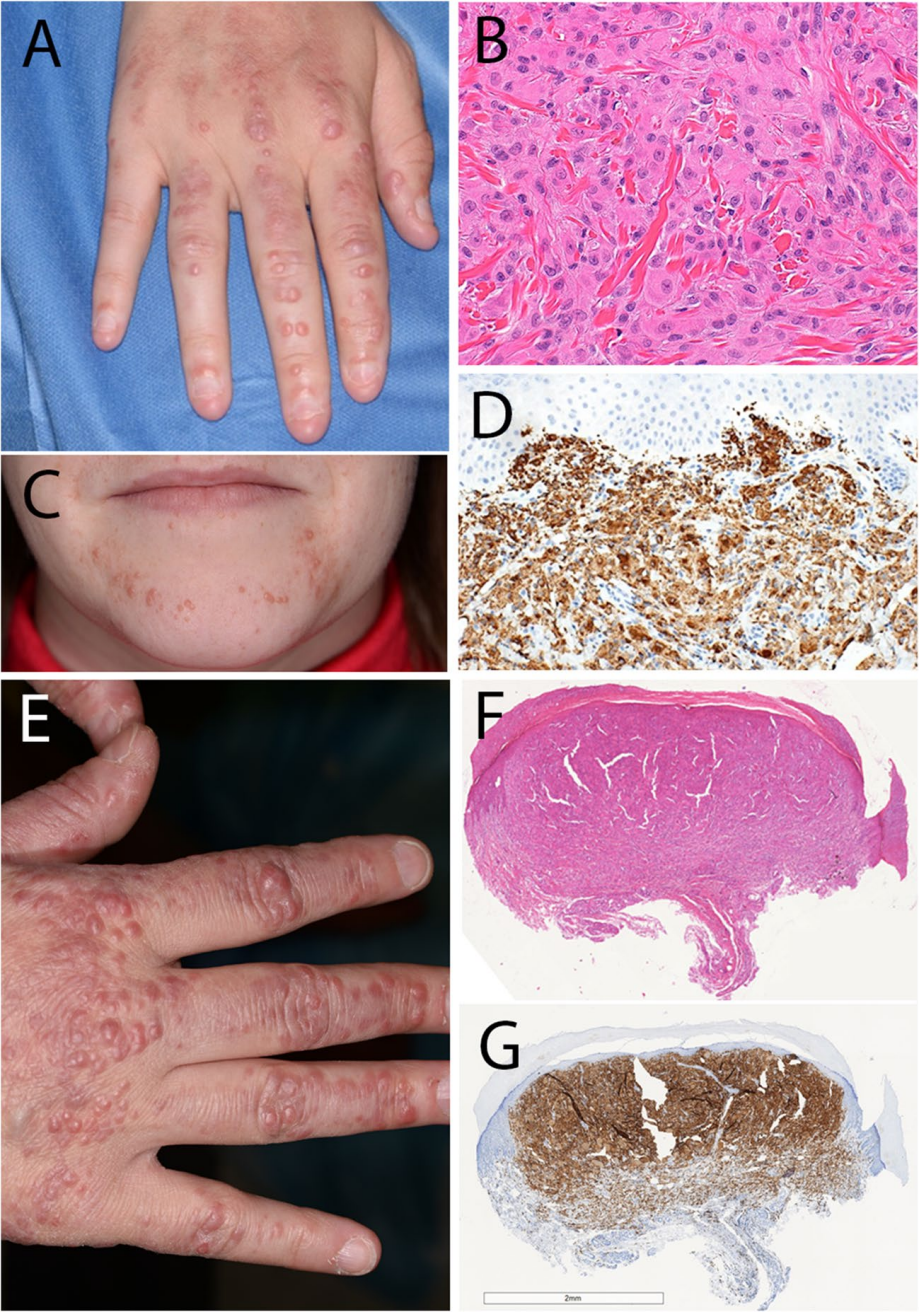
Multicentric reticulohistiocytosis. **A**–**D** Case 221,374 (J. Goodlad). **A**, **C** Typical symmetrical papules on hands and chin. **B** Proliferation of monotonous histiocytes with pink cytoplasm. **D** Strong expression of CD68. E–G. Case 214,279 (A. Bonometti). Typical reddish papules on dorsum of hand. **F** Nodular aggregate of histiocytes with **G** strong CD163 expression

Two typical cases of multicentric reticulohistiocytosis were submitted to the workshop (Fig. 5). Case 221,374 (J. Goodlad) presented in a 30-year-old female with multiple sclerosis and lesions involving both hands, forehead, and chest; at the same time, arthralgias involving both hands, as well as large joints developed. The patient responded to steroids, and the cutaneous lesions gradually disappeared. Case 214,279 (A. Bonometti) was a 27-year-old female who had a history of characteristic cutaneous lesions for several years and developed severe arthralgias during pregnancy. Both patients showed similar histological features with dense dermal accumulations of histiocytes with abundant eosinophilic cytoplasm and conventional macrophage phenotype.

### Rosai-Dorfman-Destombes disease (RDD)

Rosai-Dorfman-Destombes disease (RDD) was originally described in children as massive, usually cervical lymphadenopathy with sinus histiocytosis, that typically pursued a benign, self-limiting clinical course. Subsequently, RDD was observed in an array of extranodal locations, including skin, CNS and dura, the head and neck region and bone, among others [66–68]. Although initially thought to represent a reactive proliferation of histiocytes, mutations in MAPK pathway genes have been described in 30–50% of cases, as well as an occasional association with myeloid disorders in adults [17, 66, 69, 70]. It is currently unknown, whether both reactive and clonal forms of RDD exist, or if RDD represents more of a morphological pattern in various disorders. Despite these somehow ambiguous data, RDD has been designated a neoplastic disorder in the 5 th Edition of the WHO classification [22].

RDD is characterized by the accumulation of S100 positive histiocytes with large nuclei, prominent nucleoli and abundant cytoplasm within expanded lymph node sinuses and which show emperipolesis mostly of lymphoid cells. Additional helpful immunophenotypical features are the expression of OCT- 2, Cyclin D1, and PU.1 by the lesional cells, which however, are shared with subsets of other histiocytoses [68, 69, 71, 72]. The background population is rich in lymphocytes and plasma cells, making separation from indolent lymphomas and chronic inflammation difficult. Furthermore, many cases show increased IgG4-positive plasma cells, but the relation, if any, of RDD and IgG4-related disease remains currently unresolved.

**Fig. 6:**
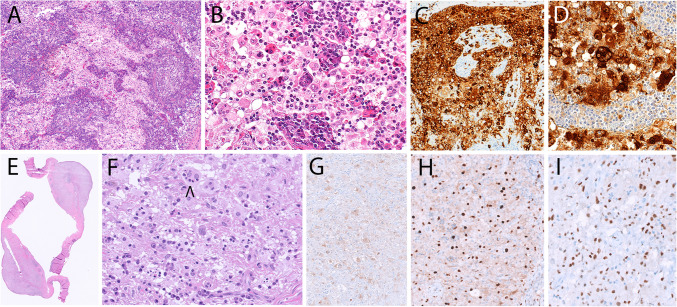
Rosai Dorfman Destombes disease (RDD). **A**–**D** Case 220,922 (P. Kusters) RDD in the setting of RAS-associated autoimmune leukoproliferative disorder in a 10-month-old male. **A**, **B** Lymph node with distended sinuses filled by histiocytes with pale cytoplasm and evidence of emperipolesis. **C** The histiocytes show strong CD163 and D. S100 expression. **E**–**I** Case 214,388 (A. Bonometti). RDD of the dura. **E** Low power view shows a nodular thickening of the dura. **F** Infiltrate of large histiocytes with frequent emperipolesis (arrowhead) and admixed lymphocytes and plasma cells. **G** Weak S100 staining of histiocytes. Strong nuclear expression of **H** Cyclin D1 and I Oct2

A total of 10 cases of RDD were submitted to the workshop, with a broad age range and diverse clinical manifestations. Case 220,922 (P. Kusters) in a 10-month-old infant with RAS-Associated Autoimmune Leukoproliferative Disorder (RALD) was an example of typical RDD in a lymph node biopsy in the background of an underlying congenital disorder (Fig. 6A–D) [73]. RDD has been described in a variety of congenital syndromes with dysregulation of immunity, including autoimmune lymphoproliferative syndrome (ALPS), and germline mutations of *SLC29 A3* encompassing Faisalabad histiocytosis and H syndrome [74]. Five submitted cases presented with dural or CNS involvement, highlighting the relatively common involvement of these sites by RDD. Case 214,388 (A. Bonometti), a morphologically and immunophenotypically classic case with involvement of dura and lung showed a *KRAS* mutation at an allelic frequency of only 2.8% (Fig. 6E–I). Molecular studies of limited scope were performed only in a subset of RDD workshop submissions, precluding further conclusions on clonality in RDD. In 3 cases, the panel felt there was insufficient evidence for a firm diagnosis of RDD due to a predominant lymphoplasmacellular infiltrate and a paucity of characteristic cells, reflecting the frequently difficult differential diagnosis in the absence of molecular alterations and the occurrence of RDD-like changes in a variety of disorders, including infections, lymphoma, IgG4-related disease, and other histiocytoses.

### ALK-positive histiocytosis

ALK + histiocytosis was initially described as a multisystem disease in three infants in which there was a proliferation of histiocytes expressing the ALK protein; a *TPM3::ALK* translocation was identified in one patient [75]. Subsequent case studies expanded the clinicopathological spectrum and demonstrated that a *KIF5B::ALK* fusion was the most common alteration, occurring in 70% of patients with ALK rearrangement [76]. The largest study of ALK + histiocytosis to date assembled 39 patients [77]. Three major groups of patients were discerned: (1a) infants with multisystem disease, including involvement of liver and hematopoietic system, (1b) children and adults with multisystemic disease without involvement of hematopoietic system and (2) patients with single system disease. CNS involvement was present in more than half of the patients from groups 1b and 2. Of note, group 2 also contained cases of solitary skin or soft tissue lesions. Separation of solitary skin ALK + histiocytosis from cases described as ALK-rearranged epithelioid histiocytoma still needs to be defined [78].

ALK + histiocytosis shows a broad morphological spectrum, ranging from classic JXG-type morphology to more spindled and epithelioid forms with less lipidized cytoplasm. Nuclear features are variable, and mild atypia can occur. Of note, some studies have reported “systemic ALK + JXG,” highlighting that terminology is still evolving. RDD-like features with emperipolesis and S100 expression is common, as are OCT- 2, CyclinD1 and phospho-ERK expression [77]. ALK staining patterns are highly variable. Of note, rare cases with nuclear ALK staining in the absence of ALK translocations have been described. Given the broad range of clinical presentation and morphology, separation of ALK + histiocytosis from ALK-rearranged soft tissue tumors such as inflammatory myofibroblastic tumors with a high number of reactive histiocytes can be a challenge.

**Fig. 7:**
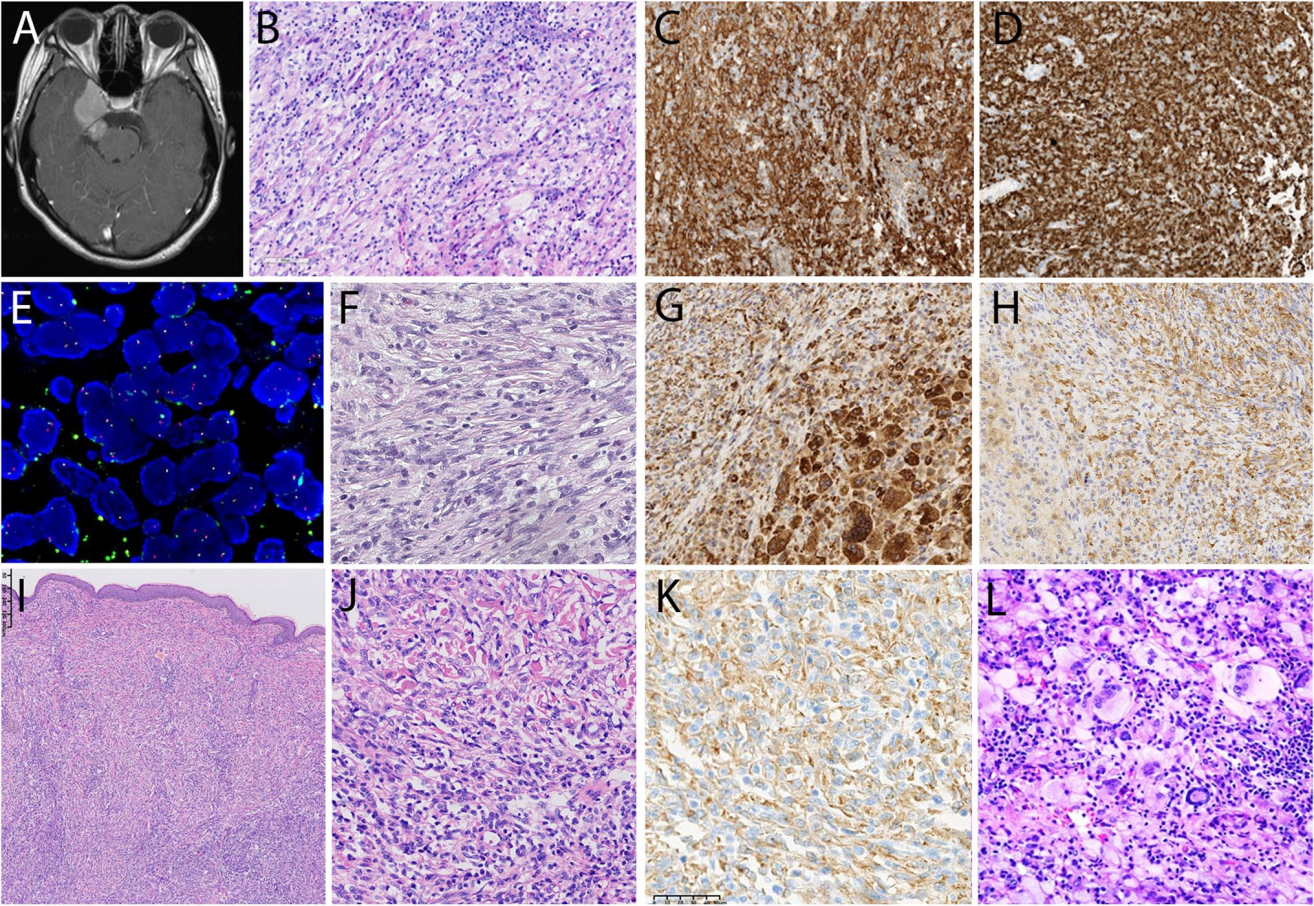
Morphological spectrum of ALK + histiocytosis. **A**–**E** Case 214,828 (S. Yao), localized ALK + histiocytosis of the CNS in a 14-year-old girl. **A** MRI reveals a mass above the petrous bone. **B** Proliferation of histiocytes with foamy cytoplasm and admixed lymphocytes. **C** Expression of CD163 and **D** ALK with the D5 F3 antibody. **E** Detection of ALK break by FISH. **F**–**H** Case 221,320 (E. Olmeda), solitary subcutaneous ALK + histiocytoma. **F** The circumscribed lesion showed both spindled (shown here) and epitheloid areas. **G** Strong reactivity for CD68 and H. ALK positivity with D5 F3 antibody. **I**–**K** Case 221,565 (W.J. Feng) 10-month-old child with skin lesions, multifocal involvement of soft tissues and inner organs. **I**, **J** Cutaneous proliferation of frequently spindled histiocytes with **K** ALK positivity. **L** Case 213,279 (Y. Zhang). Three-year-old female with intraspinal ALK + histiocytosis with cells with foamy cytoplasm and frequent multinucleation

Six cases of ALK + histiocytosis were submitted to the workshop, the age ranged from 10 months to 30 years. Three patients showed CNS involvement, similar to published data (Fig. 7A–E). Case 221,565 (J. Wu) was a 10-month-old infant with multisystemic involvement (Fig. 7I–K), and 221,320 (E. Olmeida) presented as solitary subcutaneous tumor (Fig. 7F–H). All cases exhibited an ALK rearrangement, and all 4 cases with RNA sequencing performed identifed an *KIF5B::ALK* fusion. Case 220,918 (Y. Yang) presented as large mesenteric mass with an accompanying indolent T-lymphoblastic proliferation.

## Lessons learned and diagnostic recommendations

Given the specific nature of case-based workshops, the submissions were enriched in cases with unusual clinical, morphological, or phenotypical features that do not represent a normal distribution. The study of borderline cases, however, allowed to revisit diagnostic criteria and identify areas requiring further studies.

### HLH

Distinction between familial and secondary HLH is critical because successful treatment of S-HLH depends on the underlying disorder. Hematologic neoplasms, in particular T-cell lymphomas, are the most common type of HLH-associated malignancies. Therefore, an underlying lymphoma must always be excluded from the differential diagnosis, even in pediatric cases.

There is an overlap between EBV-associated HLH and systemic EBV-positive T-cell lymphoma of childhood (SETCL). An abnormal karyotype is indicative of SETLC.

### Proliferation of reactive histiocytes

This occurs in a variety of clinical settings such as infections including vaccination and immune dysregulation. Importantly, some non-histiocytic neoplasms can specifically attract histiocytes (“landscape effect”) thereby mimicking a true histiocytosis and/or masking the underlying neoplasm.

### Phenotypic plasticity

As evidenced from multiple cases, morphological and immunophenotypical variability of lesional cells in histiocytic disorders is high, and patients may present with features fulfilling diagnostic criteria for different disorders in different sites or at different time points. The most common combination is ECD and LCH in adults. Since these discordant lesions usually are clonally related, a diagnosis of “mixed histiocytosis” with mention of the encountered lines of differentiation is a reasonable approach.

### Molecular studies

should be performed in all cases of histiocytosis with the exception of low-risk, single site disease with classic diagnostic features. Due to the high frequency of the *BRAF*^V600E^, as targeted approach may be employed first, followed by NGS if *BRAF* remains unmutated. Due to the frequently low variant allele frequency, a diagnostic sensitivity < 5% needs to be achieved. Of note, weak staining of lesional histiocytes by BRAF^V600E^-specific immunohistochemistry lacks diagnostic specificity for the *BRAF*^V600E^ mutation and should be molecularly confirmed.

### ALK + histiocytosis

shows high clinical and morphological variability and may imitate virtually any non-Langerhans cell histiocytosis. Immunostaining for ALK, preferably using the 1 A4 antibody, followed by FISH or RNA-sequencing for confirmation of an *ALK* fusion is therefore mandatory for non-LC histiocytosis, maybe with the exception of solitary skin lesions and classic JXG. Distinction from ALK-rearranged soft tissue tumors can be a challenge.

### Histiocytic disorders in association with myeloid/lymphoid disorders

Histiocytoses in adult patients are frequently associated with myeloid disorders or clonal hematopoiesis, also reflected in the presence of myeloid-type mutations. In some studies, phenotypic evolution to histiocytosis was associated with the acquisition of MAPK pathway mutations, which may serve as trigger for lineage differentiation. The clinical impact and relevance for classification of associated myeloid neoplasms needs to be defined further.

## Supplementary Information

Below is the link to the electronic supplementary material.

Supplementary file1 (DOCX 26 KB)
