# Supplementary material for: Histiocytoses and reactive proliferations of histiocytes: current state of the art and evolving concepts—a report from the joint CSHP-EA4HP-SH workshop 2024, Hefei, China
Source: Virchows Arch. 2025 Apr 10;488(2):245–62. doi: 10.1007/s00428-025-04096-4 (PMC12916990; doi:10.1007/s00428-025-04096-4)
Supplement: Supplementary file 1 — Supplementary file1 (DOCX 26 KB) [file 428_2025_4096_MOESM1_ESM.docx]

**Supplemental file for Fend F. et. al.**

**Summary of submitted cases**

| **Case #** | **Submitter** | **Age/sex** | **Localization** | **Panel diagnosis** | **Molecular/genetic findings** | **Special features** |
| --- | --- | --- | --- | --- | --- | --- |
| **Reactive and paraneoplastic histiocytoses (type 1 cases)** | | | | | | |
| 214477 | Chen M. | 23/F | Multiple LNs | Vaccine-induced LAD with lymphoid and Langerhans cell hyperplasia and hemophagocytosis | Normal female karyotype  Negative T-cell receptor gamma rearrangement studies  No evidence of a *BRAF* mutation | LAD raised clinical concern for lymphoma or other malignancy |
| 220584 | Saglietti C. | 35/F | Left buttock mass | Tenosynovial giant cell tumor | *CSF1::CD101* fusion | Benign histiocytes comprise majority of cells within tumor |
| 221331 | Croci G. | 67/F | Skin | Histiocytic proliferation of undetermined significance, in association with myeloid/lymphoid neoplasm with eosinophilia and tyrosine kinase gene fusion | PB*: ETV6::SYK* fusion detected in peripheral blood  BM: 46,XX,t(9;12)(q22;p13) | BM showed typical findings of tyrosine kinase associated myeloid neoplasm with eosinophilia. Endometrial biopsy contained aggregates of eosinophils |
| 221497 | Li P. | NP/F | Left renal mass | Unicentric Castleman disease (HV type) with indolent T-lymphoblastic proliferation | Negative T-cell receptor beta, delta and gamma rearrangement studies | Focal epithelioid stromal cell proliferation |
| 190408 | Henrich R. | 43/F | Left tibia | Xanthogranulomatous osteomyelitis |  | Clinical and radiologic findings suspicious for malignancy |
| 212247 | Li Y. | 65/F | Multiple lung nodules | Crystal-storing histiocytosis | None | No associated LPD |
| 214325 | Ge X. | NP | Lung; left main bronchus | Crystal-storing histiocytosis associated with MALT lymphoma | Clonal *IGH* and *IGK* rearrangements present |  |
| 214900 | Johnson E. | 57/M | BM | Crystal-storing histiocytosis associated with multiple myeloma | *CCND1::IGH* fusion detected by FISH | Associated MDS |
| **Hemophagocytic lymphohistiocytosis (HLH) and related disorders (type 2 cases)** | | | | | | |
| 215254 | Chen Z. | 2/F | Right cervical LN | HLH, primary EBV infection | Clonal T-cell receptor rearrangement detected  Negative HLH NGS panel | Together with Case 221571, highlights the diagnostic challenges in distinguishing between EBV-associate HLH and SETLC |
| 215644 | Hossein-Zadeh Z. | 79/F | BM | HLH associated with diffuse large B-cell lymphoma |  |  |
| 221571 | Huang J. | 3/M | Multiple LNs | HLH associated with SETLC | Clonal T-cell receptor rearrangement detected  Negative HLH NGS panel | Together with Case 215254, highlights the diagnostic challenges in distinguishing between EBV-associate HLH and SETLC |
| 221618 | Sirotnikov S. | 2mos/M | BM, splenomegaly | Primary HLH | Biallelic *STXBP2* mutations | Classic clinical and pathologic features of primary HLH |
| 221712 | Klimkowska M. | 17/F | BM | Primary HLH | Biallelic *STXBP2* mutations | Late onset primary HLH, possibly due to hypomorphic *STXBP2* mutation  Subsequent development of classic Hodgkin lymphoma |
| 224361 | Tangnuntachai N. | 66/M | BM | B-cell lymphoma (probably LPL); BM findings suspicious for HLH | No genetic studies performed | Unclear whether HLH criteria satisfied |
| 224362 | Tangnuntachai N. | 34/M | BM | HLH secondary to dengue |  | Unclear whether HLH criteria satisfied |
| 224430 | Bulterys P. | 70/M | Liver, BM | HLH secondary to disseminated tuberculosis | Liver: positive Mycobacterium tuberculosis PCR  BM: trisomy 8 by karyotyping; NGS detected mutations in *ASXL1*, *IDH2* and *CSF3R* | Concurrent MDS |

**Histiocytoses (Type 3 cases)**

| **Case #** | **Submitter** | **Age/sex** | **Localization** | **Panel diagnosis** | **Molecular findings** | **Special features** |
| --- | --- | --- | --- | --- | --- | --- |
| **Langerhans cell histiocytosis (LCH)** | | | | | | |
| 215643 | Ni X. | 61/M | LN | LCH | n.d. | associated with metastasis of nasopharyngeal carcinoma |
| 216102 | Hu X. | 28/F | CNS | LCH | *BRAF* VE1 neg. | in chordoid meningioma |
| 218720 | Dai M. | 49/M | Lymph node | LCH | n.d. |  |
| 220706 | Xiong Y. | 50/M | Kidney | LCH | *BRAF* V600E detected in both lesions | in renal cell carcinoma |
| 221278 | De Pew S. | 68/M | Bone multifocal, hypophysis | Favor mixed histiocytosis (LCH and Erdheim Chester disease) | *TP53* R282Q (c.845G>A) *TP53* P278S (c.832C>T)  *PTEN* R173C (c.517C>T) | Diabetes insipidus, multifocal osteolytic and sclerotic bone lesions over years |
| 221573 | Saglietti C. | 39/M | Multifocal bone, multiple LN, aneurysm of left brachial artery | LCH | *MAP2K1* c303_308del; p.Glu102_Ile103del | Post renal transplant; vascular involvement rare |
| 222393 | Li Z. | 60/F | Bone (skull) | LCH | n.d. | Classic bone involvement |
| **Indeterminate cell histiocytosis (IDCH)** | | | | | | |
| 214287 | Bonometti A. | 4 mo/F | Skin, several lesions on face and prox. limbs | IDCH, congenital | *ALK, BRAF, KRAS, MAP2K1, NRAS, PIK3CA* wild type | Self-healing over months |
| 215968 | Bonometti A. | 81/F | Colon, isolated tumor | IDCH | *JAK2* V617F  *KRAS* K117N | Primary myelofibrosis for 6 years, hydroxyurea therapy |
| 220829 | Laczko D. | 72/F | LN, multiple | IDCH | *BRAF* p.V600E  *SRSF2* p.P95H  *ASXL1* p.L775*  *STAG2* p.W1038* | MDS with trisomy 8, LN with nodular indeterminate cell aggregates, with low prolife-ration, rims of CD34+ blasts |
| 221640 | Li H. | 54/F | Skin, multifocal tumors | IDCH | *NCOA2* (8q13) translocation by FISH  *BRAF* c.1834 C>T p.Q612* | Leonine facies, transient response to vincristine |
| **Erdheim Chester disease (ECD)** | | | | | | |
| 218649 | Sidhu J. | 43/M | Massively thickened omentum, long bones | ECD | *BRAF* V600E  *KMT2B (MLL4)* R1083* | Associated with MDS EB-1, with 5q-, 7q- |
| 221386 | Soliman D. |  | Bone multifocal, BM, retroperitoneum, skin | ECD | *BRAF* V600E | PET-CT: retroperitoneal fibrosis, sclerotic foci in pelvic & long bones, BM and infiltration of perinephric fat |
| **Mixed histiocytosis** | | | | | | |
| 213913 | Cheng C.L. | 60/M | Bone multifocal, hypophysis, retroperitoneum | ECD and LCH | *BRAF* V600E not detected | bilateral symmetric sclerotic lesions in distal femur, tibia (ECD), lytic sternal lesion (LCH), perirenal fat and adrenal glands |
| 214289 | Bonometti A. | 62/M | Bone, skin, mucosae, hypophysis, multifocal | LCH, ECD and RDD | *MAP2K1* mutation in all lesions and BM | Diabetes insipidus, LCH and RDD in skin biopsies, ECD later in femur biopsy; progression to AML after 4 years |
| 216500 | Bonometti A. | 10/M | Skin, solitary lesion | LCH and RDD | *BRAF, MAP2K1, KRAS, NRAS, PIK3CA* wild type | Cd1a- foam cells in deeper areas |
| 221365 | Goodlad J. | 46/F | Skin, LN, soft tissues, bone multifocal | ECD and LCH | *BRAF* V600E | bony sclerosis in both distal femora and tibiae, additional LCH foci in skin and LN |
| **Juvenile Xanthogranuloma (JXG) and similar lesions** | | | | | | |
| 213991 | Cheng J. | 12/M | Skin multifocal, liver, spleen, BM | Disseminated JXG in patient with B-ALL | ALL: *CRLF2* rearranged, *CDKN2A* del.  *KRAS* G12D in follow up BM, in skin biopsy additional *BRAF* G469R and *MTOR* S2215Y | B-ALL, *BCR-ABL1*-like, development of skin lesions and HLH-like picture after ALL treatment |
| 221412 | Fan Z. | 37/M | CNS, multifocal | Non-LC histiocytosis | No MAPK pathway mutations, ALK- | JXG-like morphology/phenotype |
| 221572 | Yang S. | 8/M | CNS, multifocal, intraventricular | Non-LC histiocytosis | *BRAF* V600E | JXG-like morphology/phenotype |
| 217010 | Zhang W. | 64/M | Soft tissues, skin, bone multifocal | Non-LCH histiocytosis (ECD?) | *BRAF, KRAS, NRAS* wild type | long history of tongue cracks, coalescing brown skin lesions |
| **(Multicentric) reticulohistiocytosis (MRH)** | | | | | | |
| 214279 | Bonometti A. | 27/F | Skin multifocal | MRH | Wild type for all investigated genes | itchy papular eruption involving juxta-articular areas of hands, ear lobes; prximal exteremities; development of diffuse progressive arthralgias with severe functional impairment in pregnancy |
| 221374 | Goodlad J. | 30/F | Skin multifocal | MRH | n.d. | multiple sclerosis, 4-month history of papular, dull, red brown skin lesions affecting face, upper anterior chest, hip and the dorsa of both hands, including the fingers; multifocal arthritis |
| 215214 | Bonometti A. | 46/M | Skin, solitary lesion | Solitary reticulohistiocytoma | n.d. | Histiocytes with “ground glass” cytoplasm |
| **Rosai Dorfman Destombes disease (RDD)** | | | | | | |
| 214388 | Bonometti A. | 59/M | Solitary CNS lesion / lung multifocal | RDD | *KRAS* c.351A>C, p.K117N, 2.8% VAF |  |
| 214824 | Jin T. | 80/M | Omentum | RDD ? – difficult to classify based on available data | *NF1* c.3290A>T, VAF 3.8% | Dramatic response to Siltuximab, presence of S100+, OCT2+, CyclinD1+ macrophages |
| 214984 | Jiang L. | 67/M | CNS/Meninges multifocal | RDD | No mutations with limited panel |  |
| 216668 | Bonometti A. | 7/F | nasal sinuses, multiple sites | RDD | No mutations with 35 gene panel | Progressive involvement of bone, cranial structures, LN |
| 220560 | Harris J & Caponetti G | 63/F | CNS; LN and BM with T cell proliferation | RDD ? – difficult to classify based on available data | *KMT2D* p.A4594Gfs*12; c.13780dup; VAF: 11%; detection of T cell clonality | Infiltrate dominated by lymphocytes, typical macrophages infrequent |
| 220827 | Laczko D. | 42/M | Meninges | RDD | No mutations with lymphoma panel | Initially thought to represent lymphoma, no evidence for lymphoma w. extensive workup |
| 220922 | Kusters P. | 10mo/M | LN, spleen, liver, cytopenia | RDD in background of RAS-associated autoimmune leukoproliferative disorder (RALD) | *KRAS* c.38G>A, p.Gly13Asp, VAF 8% (BM) | Intermittent fever, rash, hepatosplenomegaly and LAP, recurrent illness episodes, well 8 y after diagnosis |
| 221455 | Zhang Q. | 50/M | Intraspinal T6-11, paravertebral | RDD | *BRAF* wild type, polyclonal for IGH | Presentation with progressive neurological symptoms, pain and difficulties walking |
| 221705 | Ye Y. | 51/F | Meninges multifocal, spinal canal | RDD (with lymphatic hyperplasia / MALT-ly?) | *KRAS, NRAS, BRAF* and *P1K3CA* wild type, no clonal B cells | History of B cell lymphoma; dominance of B cells in infiltrate |
| 222627 | Ng S.B. | 63/M | Multiple cervical, thoracic, abdominal LN | RDD | n.d. | History of chronic kidney disease, low C3 and C4 complements, anemia, increased IgG4 plasma cells |
| **ALK+ Histiocytosis** | | | | | | |
| 213729 | Zhang Y. | 3/F | Spinal cord T6-7, epidural, single lesion | ALK+ histiocytosis | ALK break by FISH | JXG-like morphology, S100+, Cyclin D1+, Oct2+ |
| 214828 | Yao S. | 14/F | Solitary CNS lesion | ALK+ histiocytosis | ALK break by FISH | Double vision and right eye abduction, trigeminal Schwannoma by imaging |
| 220918 | Yang, Y. | 19/F | Large mesenteric mass | ALK+ histiocytosis | ALK break by FISH, 52 gene panel wild type, no T cell clone | Associated with indolent T-lymphoblastic proliferation |
| 221279 | Ravindran A. | 30/M | CNS, cavernous sinus, single lesion | ALK+ histiocytosis | *KIF5B::ALK* gene fusion on RNA sequencing | Radiology: meningioma?  S100+, initially thought to represent RDD |
| 221320 | Olmeida E. | 2/F | Subcutis, solitatry lesion | ALK+ histiocytosis | *KIF5B::ALK* transcript detected | “ALK+ histiocytoma“ |
| 221565 | Feng W.J. | 10mo/? | Skin/inner organs/soft tissue multifocal | ALK+ histiocytosis | *KIF5B::ALK* transcript detected | Initial presentation with skin lesion, no follow-up provided |

BM, bone marrow; F, female; HLH, hemophagocytic lymphohistiocytosis; LAD, lymphadenopathy; LN, lymph node; M, male; LPL, lymphoplasmacytic lymphoma; MDS, myelodysplastic syndrome; NGS, next generation sequencing; NP, not provided; PB, peripheral blood; SETLC, systemic EBV-positive T-cell lymphoma of childhood
